# The correlation between serum MHR and NLR and the severity of coronary lesions in NSTE-ACS patients of different genders

**DOI:** 10.3389/fcvm.2024.1469730

**Published:** 2025-01-14

**Authors:** Yanping Yang, Xiaobing Lv, Kai Tan, Kai Li, Shaohua Li, Xia Meng, Yunyun Chen, Fuqing Wang, Hui Xin

**Affiliations:** ^1^ Department of Emergency, Qingdao West Coast New Area Central Hospital, Qingdao, Shandong, China; ^2^ Department of Cardiovascular Medicine, Affiliated Hospital of Qingdao University, Qingdao, Shandong, China

**Keywords:** non ST-elevation acute coronary syndrome, monocyte/high-density lipoprotein cholesterol ratio (MHR), neutrophil to lymphocyte ratio (NLR), SYNTAX score, receiver operating characteristic (ROC)

## Abstract

**Background:**

To study the relationship between the monocyte/high-density lipoprotein cholesterol ratio (MHR) and neutrophil-to-lymphocyte ratio (NLR) and coronary artery stenosis in Non-st-elevation acute coronary syndromes (NSTE-ACS) patients of different genders.

**Methods:**

A total of 253 control and 800 NSTE-ACS patients were included, and clinic data (29 items) were also collected. NSTE-ACS patients were divided into low-risk (0–23) and high-risk (≥ 23) groups based on the Synergy between PCI with Taxus and Cardiac Surgery (SYNTAX) score. Then, Spearman correlation and multivariate logistic regression analyses were performed to study the associated factors of high-risk SYNTAX score in male and female NSTE-ACS patients, respectively. Finally, the receiver operating characteristic (ROC) curve was used to calculate the diagnostic value of MHR and NLR for predicting high-risk SYNTAX scores in male NSTE-ACS patients.

**Results:**

Sixteen distinct factors differed between the high- and low-risk groups in male NSTE-ACS patients, a significantly higher number than female NSTE-ACS patients. Gout/hyperuricemia, smoking, NLR, and MHR are independent risk factors for arterial stenosis. At the same time, high-density lipoprotein and left ventricular ejection fraction (LVEF) are found to be protective factors in male NSTE-ACS patients. Fibrinogen, apolipoprotein B/A, and neutrophils are identified as independent risk factors for arterial stenosis in female NSTE-ACS patients, while LVEF and high-density lipoprotein are protective factors. Finally, combined NLR and MHR [*p* = 0.000, 95% confidence interval (CI) = 0.726–0.810] had better predictive efficacy on the degree of arterial vessel stenosis than NLR or MHR alone. The sensitivity and specificity of the ROC curve were 0.672 and 0.769, respectively.

**Conclusion:**

The combination of MHR and NLR shows potential for predicting and assessing the severity of coronary artery stenosis in male patients with NSTE-ACS.

## Background

In 2019, over 15 million deaths due to acute coronary syndrome (ACS) were reported globally, with 40% occurring in individuals under 70 years old (1). ACS includes ST-elevation myocardial infarction (STEMI), non-ST-elevation myocardial infarction (NSTEMI), and unstable angina pectoris. Due to their similar pathophysiology, unstable angina pectoris and NSTEMI are both known as non-ST-segment elevation acute coronary syndrome (NSTE-ACS), one of the most serious and deadly forms of myocardial infarction (2, 3). Compared to STEMI, NSTE-ACS has a greater likelihood of incidence and recurrence and can cause malignant arrhythmia, cardiogenic shock, heart failure, and other complications (3). Patients with NSTE-ACS have varying degrees of coronary artery obstruction and require a more heterogeneous treatment (4).

Atherosclerosis, characterized by inflammation and lipid accumulation, is an important mechanism of ACS (5). Elevated monocytes and reduced high-density lipoprotein cholesterol (HDL-C) levels are significant contributors to the development of atherosclerosis (6). In recent years, Monocyte/HDL cholesterol ratio (MHR) has been used to predict the severity of coronary artery disease (CAD) (7). A high neutrophil-to-lymphocyte ratio (NLR) indicates an intense inflammatory response. NLR has been identified as an autonomous risk factor for coronary heart disease and can predict the severity of ACS in coronary arteries (8–10). NLR and MHR can help to distinguish patients with ACS from those with stable angina, and their combined application can improve the sensitivity and specificity of ACS diagnosis (11). However, there are few studies on the role of MHR and NLR in NSTE-ACS patients.

The SYNTAX (Synergy Between Percutaneous Coronary Intervention With Taxus and Cardiac Surgery) is a pathology-based angiographic scoring system that assesses the complexity of CAD and has been widely used to quantify the severity of CAD lesions and predict adverse cardiovascular outcomes (12). Studies have found that MHR positively correlates with SYNTAX score in ACS patients (13, 14). However, Jiang et al. (15) did not find a statistically significant correlation between MHR and SYNTAX score in NSTE-ACS patients. Therefore, the correlation between MHR and SYNTAX score in NSTE-ACS patients has not been clearly determined. Furthermore, the correlation between NLR and SYNTAX score is controversial in NSTE-ACS patients. Altun et al. found that NLR was an independent predictor for the high SYNTAX rating group of NSTE-ACS patients, and NLR was significantly correlated with the severity of ACS vascular lesions as evaluated by the SYNTAX score (16). However, Maleki et al. found that in NSTE-ACS patients, NLR was correlated with the SYNTAX score, but after adjusting the Thrombolysis in Myocardial Infarction (TIMI) score, NLR had no significant predictive value for SYNTAX (17). Considering that the correlation between MHR, NLR, and SYNTAX scores in NSTE-ACS patients is controversial, it is necessary to investigate their correlation further.

This study aimed to investigate the relationship between MHR, NLR, and the degree of CAD in NSTE-ACS patients using the SYNTAX score. Similarly, to investigate the gender differences in the correlation between SYNTAX score and CAD, providing more biological indicators for predicting disease risk.

## Materials and methods

### Study population

This study was a retrospective analysis of patients admitted to the Department of Cardiovascular Medicine, Affiliated Hospital of Qingdao University between January and December 2021 who underwent Coronary angiography (CAG) examination. The study was approved by the Ethics Committee of the Affiliated Hospital of Qingdao University (QYFY WZLL 28592) and followed the Declaration of Helsinki guidelines. All patients signed a written informed consent for research.

Control group inclusion criteria: Patients that were not diagnosed with ACS presenting symptoms such as chest pain, chest tightness, and other issues after a CAG test. NSTE-ACS group inclusion criteria: (1) Patients diagnosed with NSTE-ACS according to the Guidelines for Diagnosis and Treatment of NSTE-ACS published by the Chinese Medical Association (2016). (2) Patients who underwent CAG examination and were treated in our hospital. At least two cardiovascular interventional physicians at the sub-senior level and higher determined the SYNTAX score.

Exclusion criteria: (1) People with infections, autoimmune diseases, or other stress response diseases such as recent trauma or severe ulcers; (2) Patients with malignant tumors, thyroid dysfunction, abnormal liver and kidney function, severe anemia, hypoproteinemia, malnutrition, and so on; (3) Patients with significant hematological abnormalities (white blood cell count < 3 × 10^9^/L or >20 × 10^9^/L, platelet count < 20 × 10^9^/L, and so on). Moreover, for the NSTE-ACS group, patients with a history of cardiomyopathy, pericardial disease, severe valvular heart disease, congenital heart disease, severe arrhythmia, and coronary artery bypass grafting were also excluded.

### Clinical data

The patients' history was collected and recorded after admission, including gender, age, height, weight, smoking history (smoking for more than one year, no less than one cigarette per day, or smoking cessation for less than six months), history of hypertension, cerebral infarction, diabetes, status post coronary stent implantation, coronary heart disease/heart failure, medicine use, and so on.

### Laboratory examination

A fasting venous blood sample was collected the morning after admission for blood routine and biochemical tests, including leukocytes, neutrophils, monocytes, lymphocytes, total cholesterol, triglycerides (TG), low-density lipoprotein cholesterol, HDL-C, blood uric acid, creatinine, cardiac troponin T (cTnT), cystatin C, and fibrinogen. Body Mass Index, MHR, NLR, and other indices were calculated.

### Ultrasonic echocardiogram

An echocardiogram is required before a CAG examination. The examining physician used the PHLIPS iE33 color Doppler echocardiography (Philips Medical Systems, Bothell, WA, USA) to examine the patients in various positions, including lying on their back and with a 30° and 45° left tilt to assess the structure and function of the heart and large blood vessels. Two clinicians performed the echocardiogram, and each patient's left ventricular ejection fraction (LVEF) was measured.

### Angiographic examination

CAG was conducted with the patient in a standard position. A percutaneous radial artery puncture was made, and an angiographic catheter was introduced into the openings of the left and right coronary arteries sequentially. At least two attending physicians and cardiologists observed the coronary vessels from multiple positions.

### SYNTAX score

The SYNTAX score was calculated based on the coronary angiography results according to the website (www. SYNTAXscore.com), and the scores were reviewed by at least two experienced attending physicians or above. SYNTAX score calculation: Coronary artery dominance (left coronary dominance +0 and right coronary dominance +1), right coronary artery stage (proximal +1, middle +1, and distal +1), right coronary branch (posterior descending branch +1 and posterior lateral branch +0.5), left main artery (right coronary artery +5 and left coronary artery +6), anterior descending branch (proximal branch +3.5 and middle branch +2.5), before descending branch apex (+1), angle of branch (the first pair +1, the second to +0.5), the cyclotron branch—proximal (right +1.5, + 2.5 from left), left side (+0.5 right and left +1), and the cyclotron branch—after descending (+1) (18, 19).

### Study design

**Figure 1:**
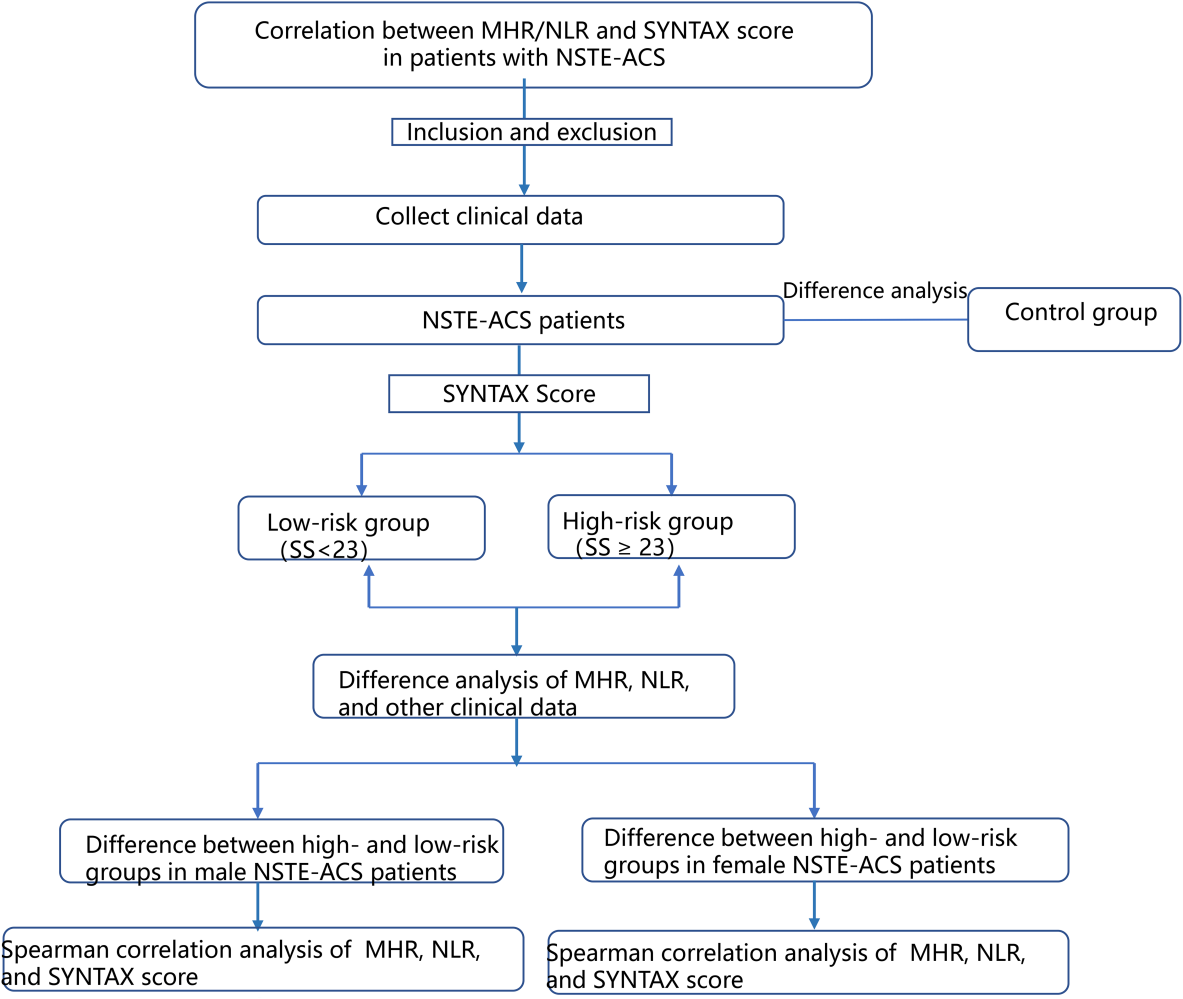
Flow chart of the study.

As shown in Figure 1, (1) the differences between the baseline data of the control and NSTE-ACS groups were first analyzed. (2) Patients in the NSTE-ACS group were divided into a low-risk group (0–23) and a high-risk group (≥23) based on their SYNTAX score, and the difference between the baseline data of the two groups was analyzed. (3) Patients with NSTE-ACS were then divided into two subgroups, male and female samples, and the difference in baseline data of SYNTAX scores of low-risk and high-risk groups in the two subgroups were analyzed, respectively. (4) The Spearman correlation analysis determined the correlation factors of the SYNTAX scores in different genders of patients with NSTE-ACS. (5) Logistic regression analysis was used to explore the related factors of SYNTAX scores for different genders. (6) The receiver operating characteristic (ROC) curve was used to calculate the diagnostic value of MHR, NLR, and their combination in the high-risk SYNTAX score of male patients with NSTE-ACS.

### Statistical analysis

All statistical analyses were performed using SPSS 20.0 statistical software (IBM Corp., Armonk, NY). Measurement data was tested for normalcy using the Shapiro–Wilk method. Data conforming to normal distribution was expressed by mean ± standard deviation else median and four-digit fraction spacing were used. The *T*-test or non-parametric (Mann–Whitney Test) was used to compare the differences between the groups. Qualitative data were presented in constituent ratios, Chi-square, or non-parametric tests to compare group differences. Correlation analysis of the two variables was performed by Pearson correlation (both variables were normally distributed) or Spearman correlation analyses. Univariate significant variables were included in multivariate logistic regression. ROC curve analysis was conducted to assess the prediction accuracy of NLR and MHR for high-risk SYNTAX scores. The appropriate threshold, sensitivity, and specificity were obtained based on the point coordinates. Bilateral *p* < 0.05 indicated a statistically significant difference.

## Results

### Comparison of the control and NSTE-ACS groups

A total of 302 participants without CAD were selected for the study, with 253 patients meeting the inclusion criteria and being included in the control group. Furthermore, 936 patients with NSTE-ACS were initially selected, and 800 met the inclusion criteria and were included in the follow-up analysis. As shown in the Supplementary Table S1, the mean age of the control group was 56.783 ± 9.793, which was significantly lower than that of the NSTE-ACS group (62.964 ± 9.055). Multiple factors in the 29 items included significantly differed between the control and NSTE-ACS groups (*p* < 0.05), such as age, sex, monocytes, NLR, LVEF, MHR, and so on.

### Comparison of the low- and high-risk groups

NSTE-ACS patients were divided into low-risk (0–23) and high-risk (≥23) groups based on the SYNTAX scores, of which 374 were in the low-risk group (188 women and 186 men), and 426 were in a high-risk group (132 women and 294 men). As shown in Table 1, gender, smoking, fibrinogen, apolipoprotein B/A, uric acid, cTnT, neutrophils, monocytes, NLR, LVEF, and MHR differed between the low-risk and high-risk groups (*p* < 0.05). The proportion of males (*p* = 0.000) and smoking history (*p* = 0.000) in the high-risk group were significantly different from those in the low-risk group.

**Table 1:** Comparison of baseline data between the low- and high-risk group based on SYNTAX score.

Variable		Low-risk (0–23, *n* = 374)	High-risk (≥23, *n* = 426)	Z/t/*Χ*^2^	*P*
Age		62.721 ± 8.907	63.178 ± 9.189	−1.613	0.107
BMI		25.801 (23.618–28.018)	25.871 (23.629–28.402)	−0.781	0.435
Gender	Female	188 (50.27%)	132 (30.99%)	30.850	0.000
	Male	186 (49.73%)	294 (69.01%)		
Hypertension	No	159 (42.51%)	167 (39.20%)	0.905	0.342
	Yes	215 (57.49%)	259 (60.80%)		
Diabetes	No	231 (61.76%)	251 (58.92%)	0.673	0.412
	Yes	143 (38.24%)	175 (41.08%)		
Hyperlipidemia	No	288 (77.01%)	316 (74.18%)	0.860	0.354
	Yes	86 (22.99%)	110 (25.82%)		
Gout/high uric acid	No	287 (76.74%)	324 (76.06%)	0.051	0.821
	Yes	87 (23.26%)	102 (23.94%)		
Status of coronary stent implantation	No	274 (73.26%)	321 (75.35%)	0.456	0.499
	Yes	100 (26.74%)	105 (24.65%)		
Coronary heart disease/heart failure	No	270 (72.19%)	302 (70.89%)	0.165	0.684
	Yes	104 (27.81%)	124 (29.11%)		
Cerebral infarction	No	283 (75.67%)	309 (72.54%)	1.016	0.313
	Yes	91 (24.33%)	117 (27.46%)		
Clinically administered drugs	No	177 (47.33%)	219 (51.41%)	1.406	0.236
	Yes	197 (52.67%)	206 (48.36%)		
Smoking	No	331 (88.50%)	297 (69.72%)	41.637	0.000
	Yes	43 (11.50%)	129 (30.28%)		
Fibrinogen (g/L)		3.150 (2.768–3.638)	3.295 (2.870–3.783)	−2.519	0.012
Triglyceride (mmol/L)		1.390 (0.900–2.225)	1.470 (1.010–2.273)	−1.097	0.273
Total cholesterol (mmol/L)		4.220 (3.465–5.238)	4.330 (3.450–5.180)	−0.252	0.801
Apolipoprotein B/A		0.742 (0.521–1.180)	0.873 (0.643–1.618)	−4.706	0.000
High-density lipoprotein (mmol/L)		1.045 (0.908–1.420)	1.150 (1.000–1.240)	−0.090	0.928
Low density lipoprotein (mmol/L)		2.415 (1.738–3.060)	2.430 (1.798–3.130)	−0.857	0.392
Creatinine (umol/L)		68.000 (56.630–78.000)	66.810 (57.000–80.000)	−0.091	0.927
Blood glucose (mmol/L)		6.095 (4.950–7.450)	6.030 (5.050–7.510)	−0.396	0.692
Uric Acid (Umol/L)		326.000 (266.790–387.103)	348.000 (283.750–411.060)	−3.510	0.000
Cystatin C (Mg/L)		0.900 (0.800–1.030)	0.930 (0.818–1.060)	−1.839	0.066
cTnT (ug/L)		0.015 (0.008–0.090)	0.080 (0.015–0.090)	−5.653	0.000
Hemameba (10^9/L)		7.060 (6.023–8.470)	7.175 (6.158–8.345)	−0.726	0.468
Neutrophil (10^9/L)		4.210 (3.358–5.253)	4.585 (3.760–5.423)	−3.533	0.000
Lymphocyte (10^9/L)		2.080 (1.630–2.463)	1.995 (1.660–2.400)	−1.176	0.240
Monocyte (10^9/L)		4.800 (3.700–5.725)	5.000 (4.000–6.200)	−2.801	0.005
NLR		2.064 (1.522–2.830)	2.520 (1.896–3.319)	−6.113	0.000
LVEF (%)		60.000 (55.000–61.000)	58.780 (52.363–60.000)	−3.653	0.000
MHR		0.429 (0.298–0.550)	0.450(0.350–0.580)	−3.027	0.02

### Comparison of the low-risk and high-risk groups of different genders of patients with NSTE-ACS

Table 2 shows that in male NSTE-ACS patients, differences in diabetes, hyperlipidemia, gout/hyperuricemia, cerebral infarction, smoking, TG, apolipoprotein B/A, HDL-C, blood glucose, uric acid, cystatin C, cTnT, monocytes, NLR, LVEF, and MHR were statistically significant between low-risk and high-risk groups (*p* < 0.05). In female patients with NSTE-ACS, differences in fibrinogen, apolipoprotein B/A, HDL-C, uric acid, cTnT, neutrophils, and LVEF were statistically significant between low-risk and high-risk groups (Table 3, *p* < 0.05). There were significantly fewer differences between the low-risk and high-risk groups in females than in males. Besides, females with high blood pressure were more likely to develop arterial stenosis than those without high blood pressure (Table 3, *p* < 0.05).

**Table 2:** Comparison of baseline data between the low- and high-risk group in male NSTE-ACS patients.

Variable		Low-risk (*n* = 186)	High-risk (*n* = 294)	Z/t/*Χ*^2^	*P*
Age		62.574 ± 8.823	62.560 ± 9.249	−0.164	0.870
BMI		25.952 (24.040–28.265)	25.989 (23.867–28.612)	−0.400	0.689
Hypertension	No	91 (48.92%)	123 (41.84%)	2.317	0.128
	Yes	95 (51.08%)	171 (58.16%)		
Diabetes	No	133 (71.51%)	181 (61.56%)	4.976	0.026
	Yes	53 (28.49%)	113 (38.44%)		
Hyperlipidemia	No	160 (86.02%)	221 (75.17%)	8.194	0.004
	Yes	26 (13.98%)	73 (24.83%)		
Gout/high uric acid	No	159 (85.48%)	221 (75.17%)	7.348	0.007
	Yes	27 (14.52%)	73 (24.83%)		
Status of coronary stent implantation	No	149 (80.11%)	221 (75.17%)	1.572	0.210
	Yes	37 (19.89%)	73 (24.83%)		
Coronary heart disease/heart failure	No	147 (79.03%)	210 (71.43%)	3.456	0.063
	Yes	39 (20.97%)	84 (28.57%)		
Cerebral infarction	No	162 (87.10%)	212 (72.11%)	14.873	0.000
	Yes	24 (12.90%)	82 (27.89%)		
Clinically administered drugs	No	95 (51.08%)	157 (53.40%)	0.287	0.592
	Yes	91 (48.92%)	136 (46.26%)		
Smoking	No	153 (82.26%)	173 (58.84%)	28.664	0.000
	Yes	33 (17.74%)	121 (41.16%)		
Fibrinogen (g/L)		3.150 (2.820–3.580)	3.260 (2.810–3.730)	−1.200	0.230
Triglyceride (mmol/L)		1.285 (0.883–1.963)	1.480 (0.990–2.320)	−2.434	0.015
Total cholesterol (mmol/L)		3.930 (3.338–4.823)	4.245 (3.335–5.140)	−1.209	0.227
Apolipoprotein B/A		0.697 (0.536–1.000)	0.827 (0.616–1.501)	−3.692	0.000
High-density lipoprotein (mmol/L)		1.420 (1.330–1.563)	1.100 (0.908–1.280)	−11.667	0.000
Low density lipoprotein (mmol/L)		2.325 (1.695–2.943)	2.395 (1.750–3.090)	−0.700	0.484
Creatinine (umol/L)		68.000 (62.000–77.000)	69.055 (59.970–81.085)	−0.335	0.738
Blood glucos (mmol/L)		5.735 (4.738–6.925)	6.005 (5.058–7.475)	−2.704	0.007
Uric Acid (umol/L)		331.500 (279.250–401.135)	355.500 (288.500–425.250)	−2.466	0.014
Cystatin C (mg/L)		0.880 (0.790–1.000)	0.930 (0.820–1.060)	−2.286	0.022
cTnT (ug/L)		0.013 (0.008–0.090)	0.050 (0.013–0.100)	−5.642	0.000
Hemameba (10^9/L)		7.050 (5.948–8.515)	7.055 (6.118–8.503)	−0.643	0.520
Neutrophil (10^9/L)		4.210 (3.458–5.378)	4.555 (3.660–5.343)	−1.802	0.071
Lymphocyte (10^9/L)		2.080 (1.628–2.475)	1.990 (1.668–2.390)	−1.141	0.254
Monocyte (10^9/L)		4.900 (3.900–6.000)	5.150 (4.300–6.300)	−2.107	0.035
NLR		1.791 (1.427–2.465)	2.600 (1.967–3.376)	−8.555	0.000
LVEF (%)		60.000 (56.000–61.000)	58.780 (52.333–60.073)	−2.992	0.003
MHR		0.437 (0.300–0.556)	0.473 (0.375–0.610)	−5.901	0.000

**Table 3:** Comparison of baseline data between the low- and high-risk group in female NSTE-ACS patients.

Variable		Low-risk (*n* = 188)	High-risk (*n* = 132)	Z/t/*Χ*^2^	*P*
Age		62.866 ± 9.011	64.448 ± 8.949	−1.684	0.092
BMI		25.459 (23.386–27.608)	25.593 (23.394–27.610)	−0.095	0.924
Hypertension	No	68 (36.17%)	44 (33.33%)	0.274	0.600
	Yes	120 (63.83%)	88 (66.67%)		
Diabetes	No	98 (52.13%)	70 (53.03%)	0.025	0.874
	Yes	90 (47.87%)	62 (46.97%)		
Hyperlipidemia	No	128 (68.09%)	95 (71.97%)	0.554	0.457
	Yes	60 (31.91%)	37 (28.03%)		
Gout/high uric acid	No	128 (68.09%)	103 (78.03%)	3.820	0.051
	Yes	60 (31.91%)	29 (21.97%)		
Status of coronary stent implantation	No	125 (66.49%)	100 (75.76%)	3.191	0.074
	Yes	63 (33.51%)	32 (24.24%)		
Coronary heart Disease/heart failure	No	123 (65.43%)	92 (69.70%)	0.642	0.423
	Yes	65 (34.57%)	40 (30.30%)		
Cerebral infarction	No	121 (64.36%)	97 (73.48%)	2.972	0.085
	Yes	67 (35.64%)	35 (26.52%)		
Clinically administered drugs	No	82 (43.62%)	62 (46.97%)	0.352	0.553
	Yes	106 (56.38%)	70 (53.03%)		
Smoking	No	178 (94.68%)	124 (93.94%)	0.080	0.777
	Yes	10 (5.32%)	8 (6.06%)		
Fibrinogen (g/L)		3.130 (2.660–3.778)	3.340 (2.950–3.858)	−2.654	0.008
Triglyceride (mmol/L)		1.630 (0.990–2.483)	1.415 (1.065–2.073)	−0.935	0.350
Total cholesterol (mmol/L)		4.445 (3.623–5.468)	4.570 (3.520–5.273)	−0.003	0.998
Apolipoprotein B/A		0.834 (0.487–1.495)	0.982 (0.744–2.170)	−3.651	0.000
High-density lipoprotein (mmol/L)		0.930 (0.820–1.000)	1.170 (1.130–1.230)	−15.232	0.000
Low density lipoprotein (mmol/L)		2.475 (1.743–3.123)	2.545 (2.005–3.250)	−1.070	0.285
Creatinine (umol/L)		65.380 (51.393–79.295)	60.000 (49.000–75.600)	−1.629	0.103
Blood glucose (mmol/L)		6.665 (5.343–8.263)	6.105 (4.990–7.550)	−1.666	0.096
Uric Acid (umol/L)		322.915 (253.185–377.458)	327.425 (277.000–381.158)	−1.315	0.189
Cystatin C(mg/L)		0.915 (0.803–1.060)	0.925 (0.790–1.068)	−0.214	0.831
cTnT(ug/L)		0.047 (0.010–0.100)	0.090 (0.020–0.090)	−2.735	0.006
Hemameba (10^9/L)		7.110 (6.090–8.333)	7.325 (6.248–8.203)	−0.416	0.677
Neutrophil (10^9/L)		4.195 (3.230–5.200)	4.655 (3.885–5.780)	−3.372	0.001
Lymphocyte (10^9/L)		2.070 (1.635–2.458)	2.015 (1.643–2.410)	−0.371	0.711
Monocyte (10^9/L)		4.650 (3.400–5.500)	4.600 (3.600–5.800)	−0.539	0.590
NLR		2.440 (1.714–3.162)	2.385 (1.779–3.223)	-.0.039	0.969
LVEF (%)		59.405 (54.325–61.000)	58.795 (52.413–60.000)	−2.171	0.030
MHR		0.410 (0.293–0.540)	0.398 (0.285–0.509)	−0.728	0.466

Upon comparing Tables 2, 3, it is evident that the presence of diabetes, hyperlipidemia, gout/hyperuricemia, and cerebral infarction significantly impact the severity of arterial stenosis in male patients with NSTE-ACS rather than in female patients. Comparing the results of biochemical tests, more factors affected the SYNTAX scores of male patients with NSTE-ACS than female patients. In male patients with NSTE-ACS, monocyte, NLR, and MHR values in high-risk groups were significantly higher than in low-risk groups (*p* < 0.05). There were no significant differences in the three values among the risk groups of female patients with NSTE-ACS.

### Correlation analysis of SYNTAX scores in NSTE-ACS patients of different genders

**Figure 2:**
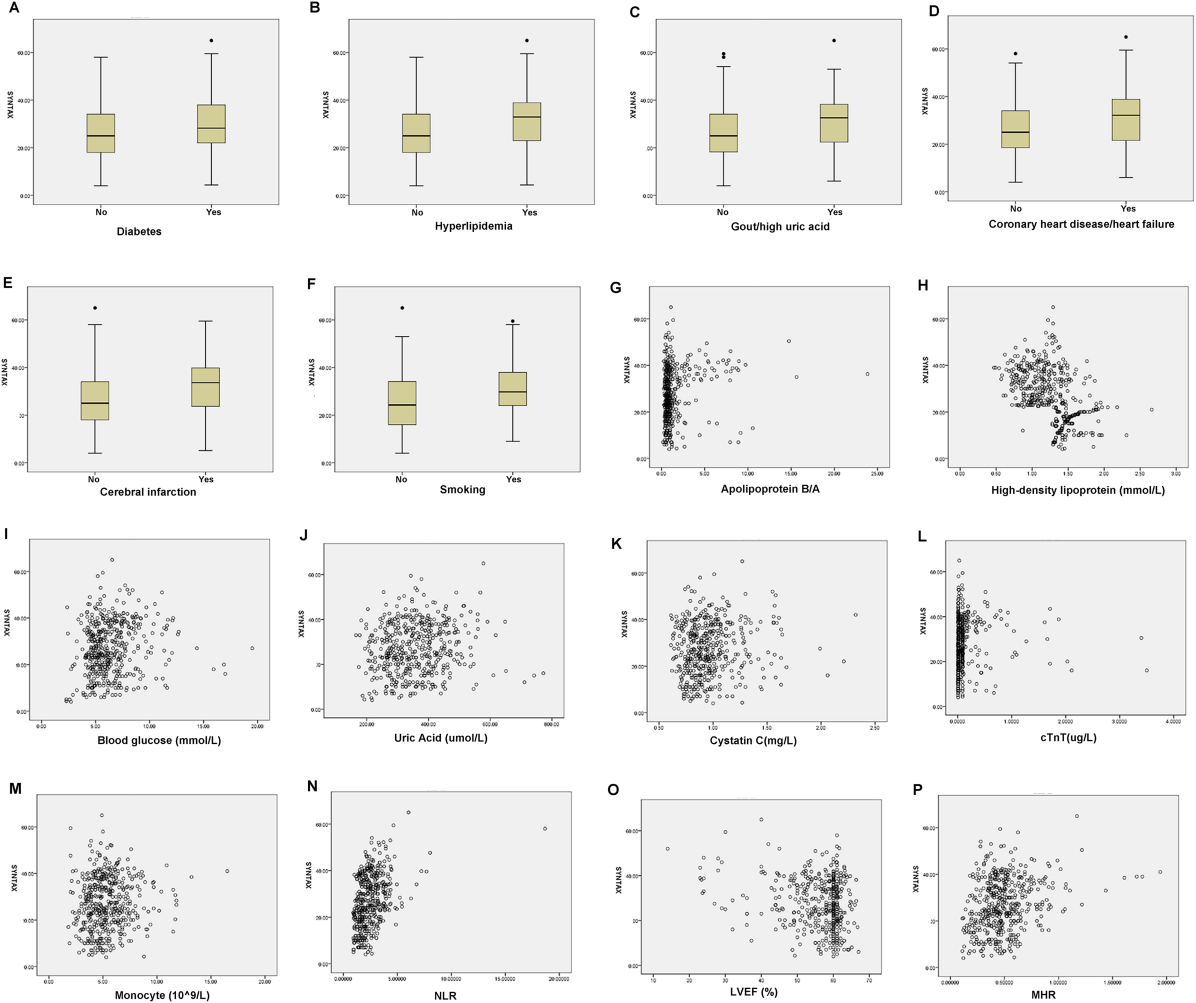
Box diagram and scatter plot of the related factors of SYNTAX scores in male patients with NSTE-ACS. **(A)** Diabetes, **(B)** Hyperlipidemia, **(C)** Gout/hyperuricemia, **(D)** Coronary heart disease/heart failure, **(E)** Cerebral infarction, **(F)** Smoking, **(G)** Apolipoprotein B/A, **(H)** high-density lipoprotein, **(I)**. Blood glucose, **(J)**. Uric Acid, **(K)**. Cystatin C, **(L)**. cTnT, **(M)**. Monocyte, **(N)**. NLR, **(O)**. LVEF, and **(P)**. MHR.

**Figure 3:**
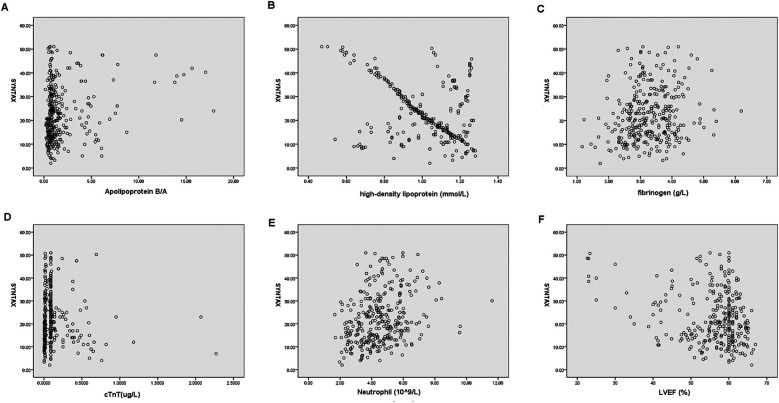
Scatterplot of the related factors of SYNTAX scores in female patients with NSTE-ACS. **(A)** Apolipoprotein B/A, **(B)** High-density lipoprotein, **(C)** fibrinogen, **(D)** cTnT, **(E)** neutrophils, and **(F)** LVEF.

As shown in Table 4, in male patients with NSTE-ACS, SYNTAX score was positively correlated with hyperlipidemia (*p* = 0.000, R = 0.178), smoking (*p* = 0.000, R = 0.240), apolipoprotein B/A (*p* = 0.000, R = 0.205), uric acid (*p* = 0.002, R = 0.138), cTnT (*p* = 0.000, R = 0.303), NLR (*p* = 0.000, R = 0.390), and MHR (*p* = 0.000, R = 0.194), indicating that the higher the values, the higher the SYNTAX scores and the degree of arterial stenosis. However, HDL-C (*p* = 0.000, R = −0.476) and LVEF (%) (*p* = 0.000, R = −0.162) were negatively correlated with SYNTAX scores, indicating that high levels of HDL-C and LVEF reduced the degree of arterial stenosis (Table 4 and Figure 2). In female patients with NSTE-ACS, SYNTAX score was positively correlated with fibrinogen (*p* = 0.002, R = 0.174), apolipoprotein B/A (*p* = 0.000, R = 0.202), cTnT (*p* = 0.004, R = 0.161), and neutrophils (*p* = 0.000, R = 0.299). However, HDL-C (*p* = 0.000, R = −0.387) and LVEF (*p* = 0.004, R = −0.159) were negatively correlated with SYNTAX scores (Table 4 and Figure 3). In summary, the SYNTAX score in male patients with NSTE-ACS had more relevant factors, so we explored the risk factors for vascular stenosis in different genders.

**Table 4:** Correlation analysis of SYNTAX score in NSTE-ACS patients of different genders.

Variable	Male		Female	
	R	*P*	R	*P*
Age	0.024	0.600	0.043	0.448
BMI	0.034	0.458	−0.035	0.531
Hypertension	0.058	0.208	−0.001	0.988
Diabetes	0.128	0.005	0.025	0.657
Hyperlipidemia	0.178	0.000	−0.027	0.632
Gout/high uric acid	0.151	0.001	−0.109	0.052
Status of coronary stent implantation	0.088	0.054	−0.101	0.072
Coronary heart disease/heart failure	0.140	0.002	−0.026	0.646
Cerebral infarction	0.210	0.000	−0.080	0.153
Clinically administered drugs	−0.039	0.400	0.020	0.717
Smoking	0.240	0.000	0.031	0.584
Fibrinogen (g/L)	0.043	0.344	0.174	0.002
Triglyceride (mmol/L)	0.143	0.002	−0.012	0.833
Total cholesterol (mmol/L)	0.057	0.216	−0.023	0.678
Apolipoprotein B/A	0.205	0.000	0.202	0.000
High-density lipoprotein (mmol/L)	−0.476	0.000	−0.387	0.000
Low density lipoprotein (mmol/L)	0.035	0.438	0.030	0.599
Blood glucose (mmol/L)	0.174	0.000	−0.072	0.196
Creatinine (umol/L)	−0.003	0.955	−0.090	0.108
Uric Acid (umol/L)	0.138	0.002	0.094	0.093
Cystatin C (mg/L)	0.125	0.006	0.007	0.902
cTnT (ug/L)	0.303	0.000	0.161	0.004
Hemameba (10^9/L)	0.039	0.390	0.068	0.226
Neutrophil (10^9/L)	0.086	0.059	0.299	0.000
Lymphocyte (10^9/L)	−0.071	0.118	−0.039	0.483
Monocyte 108	0.101	0.027	0.018	0.746
NLR	0.390	0.000	0.044	0.434
LVEF (%)	−0.162	0.000	−0.159	0.004
MHR	0.194	0.000	0.000	0.998

### Risk factors for vascular stenosis in different genders of patients with NSTE-ACS

Meaningful variables in the above correlation analysis were included in the logistic regression. Table 5 shows that gout/hyperuricemia [*p* = 0.035, OR = 2.164 (1.056–4.431)], smoking [*p* = 0.005, OR = 2.351 (1.301–4.246)], NLR [*p* = 0.000, OR = 2.400 (1.773–3.248)], and MHR [*p* = 0.004, OR = 9.430 (2.073–42.909)] were risk factors for SYNTAX score in male patients with NSTE-ACS, indicating that the higher the value, the greater the degree of arterial stenosis. HDL-C [*p* = 0.000, OR = 0.015 (0.006–0.042)] and LVEF [*p* = 0.018, OR = 0.960 (0.928–0.993)] are protective factors, and low levels of HDL-C and LVEF can prevent or alleviate arterial stenosis.

**Table 5:** Analysis of risk factors for vascular stenosis in male NSTE-ACS patients.

Variable	B	S.E.	Wald	*P*	OR (95%CI)
Hyperlipidemia	0.626	0.365	2.951	0.086	1.870 (0.916–3.821)
Gout/high uric acid	0.772	0.366	4.452	0.035	2.164 (1.056–4.431)
Smoking	0.855	0.302	8.023	0.005	2.351 (1.301–4.246)
High-density lipoprotein	−4.171	0.514	65.827	0.000	0.015 (0.006–0.042)
NLR	0.875	0.154	32.122	0.000	2.400 (1.773–3.248)
LVEF	−0.041	0.017	5.590	0.018	0.960 (0.928–0.993)
MHR	2.244	0.773	8.426	0.004	9.430 (2.073–42.909)
Constant	4.587	1.333	11.836	0.001	98.243

Table 6 shows that in female patients with NSTE-ACS, fibrinogen [*p* = 0.027, OR = 1.457 (1.045–2.033)], apolipoprotein B/A [*p* = 0.002, OR = 1.209 (1.069–1.367)], apolipoprotein B/A [*p* = 0.002, OR = 1.209 (1.069–1.367)], Neutrophils [*p* = 0.010, OR = 1.263 (1.057–1.509)] were independent risk factors for arterial stenosis. LVEF [*p* = 0.022, OR = 0.963 (0.933–0.995)] and HDL-C [*p* = 0.000, OR = 0.063 (0.015–0.264)] were protective factors for arterial stenosis.

**Table 6:** Analysis of risk factors for vascular stenosis in female NSTE-ACS patients.

Variable	B	S.E.	Wald	*P*	OR (95%CI)
Fibrinogen	0.377	0.170	4.913	0.027	1.457 (1.045–2.033)
Apolipoprotein B/A	0.190	0.063	9.184	0.002	1.209 (1.069–1.367)
cTnT	−0.944	0.736	1.646	0.199	0.389 (0.092–1.646)
Neutrophil	0.233	0.091	6.608	0.010	1.263 (1.057–1.509)
LVEF	−0.038	0.016	5.275	0.022	0.963 (0.933–0.995)
High-density lipoprotein	−2.772	0.735	14.205	0.000	0.063 (0.015–0.264)
Constant	2.008	1.351	2.209	0.137	7.447

### NLR and MHR predict the degree of vascular stenosis in male patients with NSTE-ACS

**Figure 4:**
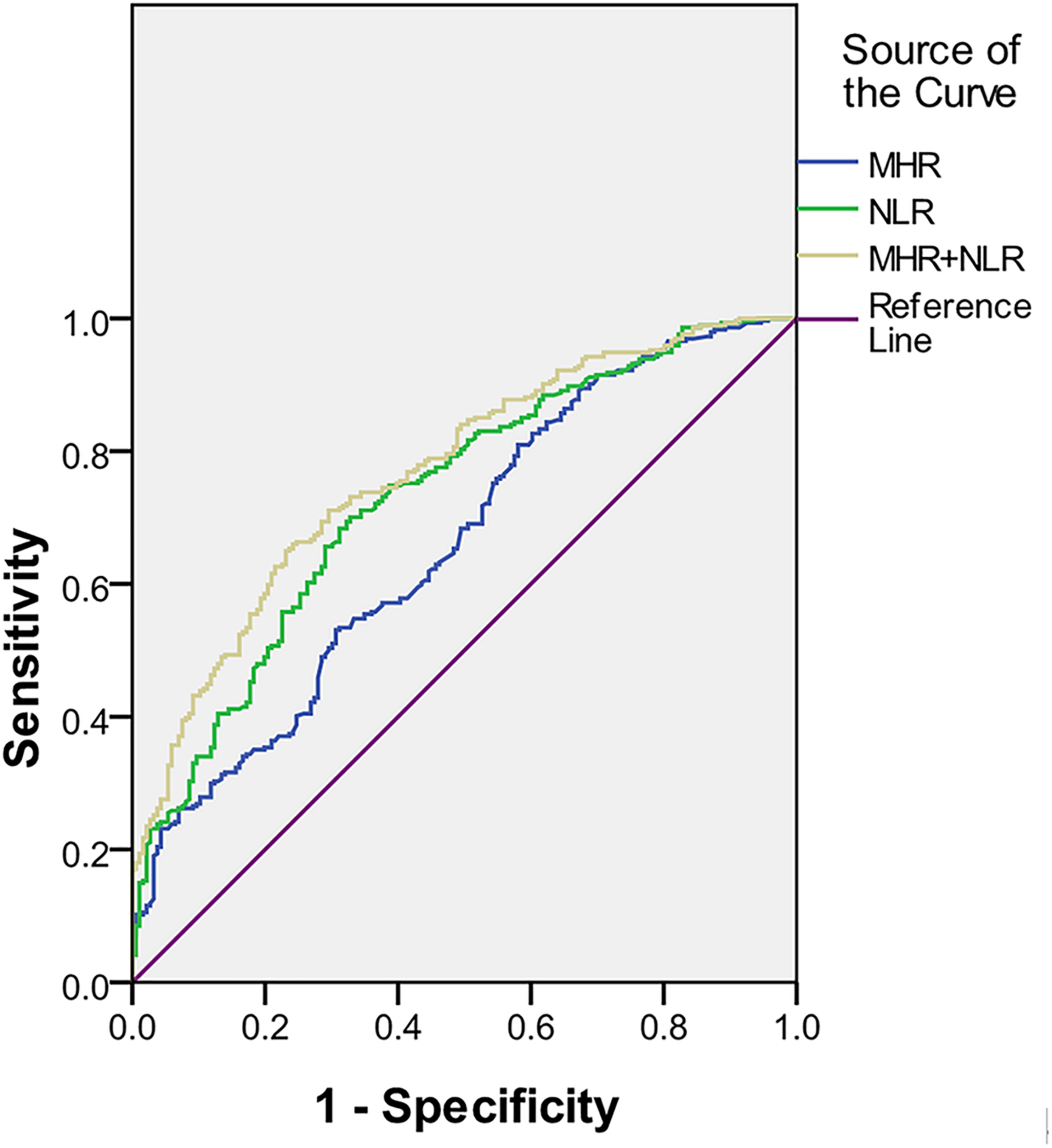
ROC curve analysis of NLR and MHR in male patients with NSTE-ACS.

The above results indicate that NLR and MHR are independent risk factors for SYNTAX scores. The ability of NLR and MHR to predict the high-risk SYNTAX scores was further evaluated. As shown in Figure 4 and Table 7, the area under the curve values for ROC analyses using NLR individually and combining NLR and MHR were 0.732 and 0.768, respectively. The optimal critical value of NLR was 2.098, and the corresponding sensitivity and specificity values were 0.701 and 0.672, respectively. The specificity of combined NLR and MHR in predicting the degree of arterial vessel stenosis was 0.769, higher than that of MHR and NLR individually.

**Table 7:** ROC curve parameters of NLR and MHR in male NSTE-ACS patients.

Variable	AUC	Standard error	*P*	95%CI	Cut-off	Sensitivity	Specificity
MHR	0.660	0.025	0.000	0.610–0.709	0.363	0.810	0.419
NLR	0.732	0.023	0.000	0.686–0.777	2.098	0.701	0.672
MHR + NLR	0.768	0.021	0.000	0.726–0.810	0.641	0.650	0.769

## Discussion

This study showed gender differences in the effects of multiple indicators on SYNTAX scores. In male patients with NSTE-ACS, gout/hyperuricemia, smoking, NLR, and MHR were independent risk factors for arterial vessel stenosis, and HDL-C and LVEF were protective factors. In female NSTE-ACS patients, fibrinogen, apolipoprotein B/A, and neutrophils were independent risk factors for arterial stenosis, and LVEF and HDL-C were protective factors for arterial stenosis. NLR combined with MHR can predict the SYNTAX score grading in male patients with NSTE-ACS.

Atherosclerotic lesions are early pathological stages of ACS. Multiple reports have shown the existence of neutrophils and neutrophil extracellular traps (NETs) in atherosclerotic lesions in both mice and humans (20, 21). NETs can damage the endothelium through the action of Interleukin 1 (IL-1) and cathepsin G and promote the expression of ICAM-1, VCAM-1, and tissue factors related to plaque thrombosis (22). Moreover, at the site of plaque rupture, neutrophils interact with platelets to promote the development of NETs, thus accelerating thrombosis (23). Lymphocytes play a vital role in the adaptive immune response and participate in the prolonged inflammatory phase of atherosclerosis (24). It has been confirmed that there are activated T lymphocytes (such as CD4 + and CD8+ T cells) in atherosclerotic plaques (25). In addition, CD8 + T cells are negatively correlated with coronary atherosclerosis and are independent predictors of acute coronary events (26). An increase in neutrophils or a decrease in lymphocytes will result in a higher NLR value, indicating a more intense inflammatory response. During inflammation, neutrophils play a crucial role in regulating the advancement of arterial wall tissue damage, while the death of lymphocytes worsens arteriosclerosis (27). This study showed that the NLR value of the high-risk SYNTAX group was significantly higher than that of the low-risk group (*p* < 0.001). Furthermore, the multivariate logistic analysis showed that NLR value was an independent risk factor for a high SYNTAX score. Consistent with other studies, Arbel et al. suggested that NLR value could predict the severity of ACS coronary artery and was an independent risk factor for CAD (10). However, this study also indicated that NLR has various effects on SYNTAX grading of NSTE-ACS patients of different genders.

MHR is a novel predictor that reflects the balance between monocytes and HDL-C, inflammation, and oxidative stress (28). MHR may better predict clinical outcomes than monocyte count and HDL-C concentration alone (29). Studies have reported that the incidence of long-term major cardiovascular adverse events (MACE) in ACS patients with elevated MHR is 1.4 times higher than in ACS patients with lower MHR (30). Acikgoz et al. (31) showed that increased MHR could predict the in-hospital mortality (HR = 3.745, 95% CI: 1.304–5.950) and 5-year mortality (HR = 2.048, 95% CI: 1.225–4.091, *p* = 0.014). Sun et al. have comprehensively analyzed eight studies (including 6,480 ACS patients) to evaluate the prognostic role of MHR in ACS patients, and the results showed that high levels of MHR were associated with an increased risk of MACE (RR: 1.65; 95% CI: 1.36–2.02) and all-cause mortality (RR: 2.61; 95% CI: 1.29–4.89), indicating that MHR can be used as a potential prognostic indicator for ACS (32). This study has found significant differences in MHR between the control and NSTE-ACS groups and between low-risk and high-risk SYNTAX score groups. In male patients with NSTE-ACS, the MHR of the patients with high SYNTAX scores was significantly higher than that of the group with low SYNTAX scores. Moreover, MHR is an independent risk factor for SYNTAX score in male patients with NSTE-ACS, which can predict the degree of arterial vessel stenosis.

The mechanism of MHR-induced vascular stenosis may be related to the following explanations. The breakdown of endothelial cells in the blood vessel wall promotes the deposition of low-density lipoprotein cholesterol and causes an oxidation reaction to produce oxidized low-density lipoprotein (Ox-LDL). Ox-LDL activates pattern recognition receptors, triggering immunological responses like the release of pro-inflammatory cytokines and the uptake of oxides by blood vessels and phagocytes, leading to atherosclerosis (33). Circulating monocytes are recruited to the inner membrane by Ox-LDL, where they transform into resident macrophages, and these macrophages consume Ox-LDL, turning into foam cells, which triggers the release of pro-inflammatory cytokines and the accumulation of adipocytes (34). However, HDL-C can clear the cholesterol in the artery wall and neutralize the pro-inflammatory and pro-oxidative effects of monocytes, showing anti-atherosclerosis effects (7). Therefore, an increase in monocyte count and a decrease in HDL-C concentration will lead to a rise in MHR and atherosclerosis.

Notably, this study also showed that SYNTAX grading in patients with NSTE-ACS of different genders was affected by various factors. Multiple studies have shown that the dynamic course of CAD significantly differs between men and women. Premenopausal women are relatively resistant to CAD, while postmenopausal women have an increased risk of CAD, comparable to a 70-year-old man (35). Moreover, the manifestations of CAD are also different between men and women. In contrast to men, the etiology of ischemic heart disease in women is predominantly attributed to pathological processes, including micro embolism resulting from erosion of active atherosclerotic plaques, abnormal coronary artery response, and microvascular endothelial dysfunction, rather than anatomical blockage of the coronary arteries (35).

There are also differences in sex hormones and immune responses between men and women. The anti-inflammatory effects of estrogen on macrophages help prevent atherosclerosis. Moreover, estrogen has been reported to promote the polarization of the immune response towards antibody-mediated humoral immunity, mainly involving the Th2 (auxiliary) and Treg (regulatory) types of CD4+ T lymphocytes (36). Estrogen stimulates B cells and enhances antibody production by B lymphocytes, whereas androgens inhibit B-cell development, decreasing antibody synthesis in humans (37, 38). Androgens are believed to stimulate cell-mediated and Th1 responses (39). So, women have an increased antibody response to infections and vaccines compared to men. In men, testosterone increases the activation of mast cells and macrophages, leading to the development of TLR2+/TLR4+/M2b macrophages and foam cells within the atherosclerotic plaques. Besides, cytokines, enzymes, and other mediators remodeled the vessel wall, resulting in a thrombus formation (38). This regulation of the immune system by androgens, combined with early exposure to behavioral factors such as smoking, early onset of hypertension, or multiple sclerosis, may lead to inconsistencies in the factors affecting the degree of vascular stenosis in patients of different genders with NSTE-ACS.Gender differences in performance, sex hormones, immune response characteristics, and risk factors of CHD patients may lead to gender differences in MHR and NLR. Pan et al. found that NLR was associated with high SYNTAX scores in men with CAD and was an independent risk factor and predictor of CAD in men but not women (40). Xu et al. found that MHR was positively correlated with SYNTAX scores in males with stable CAD, which could help interventional cardiologists detect high-risk patients before coronary artery catheterization, but its application may only be limited to men (41). Similar results were also found in this study. Only in male patients with NSTE-ACS, NLR and MHR differed significantly between low-risk and high-risk SYNTAX score groups and were independent correlated factors for high SYNTAX scores. MHR and NLR had different abilities to predict stenosis severity in CAD of different sexes, which may be related to the immune mechanism of varying sex hormones. Estrogen can act directly on monocytes, reducing their ability to migrate to the artery wall, thereby reducing the inflammatory response of arteriosclerosis. Besides, estrogen can increase T and B cells' tolerance, reducing their accumulation within the artery wall. At the same time, estrogen may also increase the number of anti-inflammatory lymphocytes (such as regulatory T cells Tregs), promote immune tolerance, and reduce arteriosclerosis inflammation (42, 43). While, testosterone is associated with an increase in the inflammatory response of immune cells, particularly the activity of monocytes and the secretion of pro-inflammatory cytokines (e.g., TNF-α, IL-6). Besides, testosterone can stimulate the activity of neutrophils, enhancing their aggregation, which further exacerbates arterial inflammation and promotes plaque formation and instability (44, 45). In summary, the different effects of sex hormones make men and women differ in immune response and progression of arteriosclerosis, which may also explain the gender-related differences in the impact of MHR and NLR on the SYNTAX scores in CAD patients.

## Conclusion

NLR paired with MHR can effectively predict SYNTAX scores in male patients with NSTE-ACS, offering a theoretical foundation for assessing coronary artery stenosis in this patient population.

## Data Availability

The original contributions presented in the study are included in the article/Supplementary Material, further inquiries can be directed to the corresponding author.
