# Supplementary material for: The correlation between serum MHR and NLR and the severity of coronary lesions in NSTE-ACS patients of different genders
Source: Front Cardiovasc Med. 2025 Jan 14;11:1469730. doi: 10.3389/fcvm.2024.1469730 (PMC11772418; doi:10.3389/fcvm.2024.1469730)
Supplement: Supplementary file 1 [file Table1.pdf]

Supplementary Table 1 Analysis of basal information between control and NSTE-ACS patients

**variable**		**NSTE-ACS group**	**control**	**Z/t/X^2**	**P**
age		62.964±9.055	56.783±9.793	9.278	<0.001
BMI		25.981±3.441	26.118±3.330	0.558	0.577
gender	female	320(40.00%)	124(49.01%)	6.401	0.011
	male	480(60.00%)	129(50.99%)		
hypertension	No	326(40.75%)	130(51.38%)	8.852	0.003
	Yes	474(59.25%)	123(48.62%)		
diabetes	No	482(60.25%)	218(86.17%)	57.930	<0.001
	Yes	318(39.75%)	35(13.83%)		
hyperlipidemia	No	604(75.50%)	247(97.63%)	60.710	<0.001
	Yes	196(24.50%)	6(2.37%)		
Gout/high uric acid	No	611(76.38%)	247(97.63%)	57.541	<0.001
	Yes	189(23.63%)	6(2.37%)		
Status of coronary stent implantation	No	595(74.38%)	253(100.00%)	80.504	<0.001
	Yes	205(25.63%)	0(0.00%)		
Coronary heart disease/heart failure	No	572(71.50%)	247(97.63%)	75.922	<0.001
	Yes	228(28.50%)	6(2.37%)		
Cerebral infarction	No	592(74.00%)	245(96.84%)	61.486	<0.001
	Yes	208(26.00%)	8(3.16%)		
Clinically administered drugs	No	396(49.50%)	133(52.57%)	0.695	0.404
	Yes	403(50.38%)	120(47.43%)		
Smoking	No	628(78.50%)	218(86.17%)	7.152	0.007
	Yes	172(21.50%)	35(13.83%)		
fibrinogen (g/L)		3.210(2.810-3.730)	2.780(2.443-3.085)	-9.750	<0.001
triglyceride (mmol/L)		1.3220(0.778-2.106)	0.880(0.670-1.263)	-1.062	0.0223
total cholesterol (mmol/L)		4.260(3.453-5.180)	4.230(3.510-4.960)	-0.176	0.861
Apolipoprotein B/A (mmol/L)		0.830(0.581-1.381)	0.590(0.480-0.730)	-10.354	<0.001
high-density lipoprotein (mmol/L)		1.130(0.940-1.330)	1.190(1.050-1.460)	-4.930	<0.001
low density lipoprotein (mmol/L)		2.425(1.753-3.090)	2.460(1.825-2.955)	-0.056	0.955
creatinine (umol/L)		67.028(57.568-79.231)	63.371(51.098-80.325)	-0.046	0.047
blood glucose (mmol/L)		6.055(5.020-7.488)	5.290(4.790-5.845)	-7.159	<0.001

Uric Acid (umol/L)	338.000(277.000-400.945)	327.000(271.000-384.000)	-1.566	0.117
cystatin C(mg/L)	0.920(0.800-1.050)	0.850(0.755-0.930)	-6.418	<0.001
cTnT(ug/L)	0.033(0.010-0.090)	0.006(0.004-0.008)	-18.840	<0.001
hemameba (10^9/L)	7.125(6.103-8.383)	6.240(5.185-7.280)	-8.150	<0.001
Neutrophil (10^9/L)	4.410(3.613-5.348)	3.590(2.895-4.400)	-8.989	<0.001
lymphocyte (10^9/L)	2.030(1.650-2.430)	1.940(1.610-2.370)	-0.840	0.401
monocyte (10^9/L)	4.900(3.900-6.000)	0.380(0.310-0.500)	-24.004	<0.001
NLR	2.330(1.697-3.133)	1.713(1.394-2.291)	-8.298	<0.001
LVEF (%)	59.175(53.838-61.000)	6<0.001(6<0.001-61.000)	-10.261	<0.001
MHR	0.440(0.323-0.561)	0.312(0.226-0.445)	-8.333	<0.001
